# Pembrolizumab and olaparib in a cisplatin-refractory testicular cancer patient with a high TMB: first case report

**DOI:** 10.1177/17562872251322648

**Published:** 2025-03-14

**Authors:** Nils C. H. van Creij, Gerald Klinglmair, Leonhard Gruber, Antonia Partl, Alain G. Zeimet, Frédéric R. Santer, Simon Schnaiter, Roman Mayr, Felizian Lackner, Zoran Culig, Andreas Seeber, Renate Pichler

**Affiliations:** Department of Urology, Comprehensive Cancer Center Innsbruck, Medical University of Innsbruck, Innsbruck, Austria; Division of Experimental Urology, Department of Urology, Medical University of Innsbruck, Innsbruck, Austria; Department of Urology, Comprehensive Cancer Center Innsbruck, Medical University of Innsbruck, Innsbruck, Austria; Department for Radiology, Medical University of Innsbruck, Innsbruck, Austria; Department of Urology, Comprehensive Cancer Center Innsbruck, Medical University of Innsbruck, Innsbruck, Austria; Department of Obstetrics and Gynecology, Medical University of Innsbruck, Innsbruck, Austria; Department of Urology, Comprehensive Cancer Center Innsbruck, Medical University of Innsbruck, Innsbruck, Austria; Division of Experimental Urology, Department of Urology, Medical University of Innsbruck, Innsbruck, Austria; Institute of Human Genetics, Medical University of Innsbruck, Innsbruck, Austria; Department of Urology, St. Josef Medical Center, University of Regensburg, Regensburg, Germany; Department of Urology, Comprehensive Cancer Center Innsbruck, Medical University of Innsbruck, Innsbruck, Austria; Department of Urology, Comprehensive Cancer Center Innsbruck, Medical University of Innsbruck, Innsbruck, Austria; Division of Experimental Urology, Department of Urology, Medical University of Innsbruck, Innsbruck, Austria; Department of Hematology and Oncology, Comprehensive Cancer Center Innsbruck, Medical University of Innsbruck, Innsbruck, Austria; Department of Urology, Comprehensive Cancer Center Innsbruck, Medical University of Innsbruck, Anichstreet 35, Innsbruck 6020, Austria

**Keywords:** case report, germ cell tumors, immunotherapy, PARPi

## Abstract

Germ cell tumors (GCTs) represent about 5% of urological cancers affecting mostly younger males with increasing incidence in the last decades. GCTs are very sensitive to cisplatin-based therapy and are highly curable regardless of metastatic stage, likely based on having inherited unique mechanisms of sensitivity to DNA damage and other stressors to prevent germline mutations. Here, we present the first case of a 60-year Caucasian male with a heavily pretreated, cisplatin-refractory extragonadal non-seminomatous GCT (choriocarcinoma) treated with pembrolizumab and olaparib based on high programmed death-ligand 1 expression (tumor proportion score 50% and a combined positive score 55%) high tumor mutational burden, borderline genomic loss of heterozygosity, and a heterozygous variant of uncertain significance in the DNA repair gene *ATM*, as confirmed by next-generation sequencing (NGS) analysis. Despite a notable decrease in b-hCG within 4 weeks after starting pembrolizumab and olaparib, b-hCG increased steadily again afterward. In addition, the patient developed an immune-related pneumonitis with a fatal outcome 3 months later. NGS with subsequent targeted treatment possibilities might present a helpful step toward precision medicine in GCT patients who have exhausted all other conventional treatment options, and genetic testing should therefore be offered to patients prior to progression.

## Introduction

In 2023, it is estimated that there were 9470 new cases of germ cell tumors (GCTs) in the United States, resulting in about 440 deaths. GCTs represent roughly 1% of all male cancer diagnoses, and it is the most common cancer entity in young males aged between 20 and 40 years.^1^ GCTs can be categorized histologically into seminomas, non-seminomas, and spermatocytic seminomas, including those with mixed features.^2^ Most commonly, seminomas or non-seminomas are present at the age of 35–39 or 25–29 years, respectively.^3^ Despite the excellent response to platinum-containing chemotherapy, 10%–15% of patients experience relapse or progression. In this group, 25%–70% can potentially be cured through salvage chemotherapy, sometimes accompanied by a surgical resection of the residual disease.^4^ However, the prognosis significantly worsens for patients who relapse after salvage therapy, with less than 10% achieving long-term responses. The development of more effective treatments for these patients faces challenges, particularly in cases with evident cisplatin resistance, and the absence of identified molecular pathways.^5^ Consequently, efforts to test new drugs in this patient population have so far been limited to small-scale phase II trials, often with negative results.^6^ Therefore, there is an unmet need for drug development in this specific subgroup of patients.^7^

The underlying molecular mechanisms of GCTs to DNA-damaging chemotherapies are not fully elucidated.^8^ It is hypothesized that the high response rates of GCTs to cisplatin are due to the low threshold for apoptosis induction in these tumor cells. Another explanation could be the reduced capacity for DNA damage repair (DDR). In particular, interstrand crosslink repair and homologous recombination repair (HRR) are frequent defective DDR pathways in GCTs.^9^ DDR inhibitors have been recently introduced in oncological disease management. Olaparib is a Poly(ADP-ribose) polymerase (PARP) inhibitor (PARPi) that shows the highest efficiency in tumor cells with HRR deficiency (HRD), with approvals in ovarian, breast, prostate, and pancreatic cancers. Treatment of patients with olaparib involves prior detection of mutations in genes of the HRR pathway, and/or by detection of the HRD-based genomic instability by assessment of loss-of-heterozygosity (LOH). As GCTs frequently show defects in HRR, it was proposed that PARP inhibition might be an efficient treatment option.^8^ Clinical trials have tried to answer this question, evaluating PARPi either as a single agent (NCT02533765) or combined with gemcitabine and carboplatin (NCT02860819).^10,11^ However, the phase II ICG-02 trial of olaparib monotherapy in patients with relapsed/refractory metastatic germ cell cancer showed marginal efficacy with progressive disease in 13 (72.2%) of 18 patients. A germline DNA repair profile panel showed only one *BRCA1*-mutated case associated with a 4-month stable disease.^11^ The GCT-SK-004 phase II trial combining gemcitabine, carboplatin, and the PARPi veliparib in patients with multiple relapsed/refractory GCTs also failed to achieve its primary endpoint with a 12-month progression-free survival in only one (6.7%) patient.^10^ Here, we first report a cisplatin-refractory non-seminomatous GCT treated with the immune checkpoint inhibitor (ICI) pembrolizumab and the PARPi olaparib based on next-generation sequencing (NGS) analysis confirming high tumor mutational burden (TMB) status, a low genomic LOH (gLOH) score, and a variant with uncertain significance in the DDR gene ATM.

## Case presentation

A 60-year-old Caucasian male with no risk factors presented with a large, palpable mass in the lower abdomen. Cross-sectional CT imaging showed a 20.0 × 12.5 cm^2^ mass in the retroperitoneum, consecutive hydronephrosis in the left kidney, and multiple bilateral pulmonary lesions. Initially, the retroperitoneal mass was misdiagnosed as a sarcoma. CT-guided biopsy of the retroperitoneal mass indicated a dedifferentiated non-small-cell carcinoma with notable high programmed death-ligand 1 (PD-L1) expression, showing a tumor proportion score of 50% and a combined positive score of 55%.

Two months after the initial presentation, the patient was referred to our urological department with notable progression in the pulmonary metastases and a new liver metastasis. At this time, testicular tumor markers were positive for beta human chorionic gonadotropin (b-hCG) at 7945.0 U/l, and lactate dehydrogenase at 1203 U/l. Alpha1-fetoprotein was negative <2.7 µg/l. Testicular sonography revealed no intratesticular tumor. Given these new findings, the previous retroperitoneal biopsy was re-examined, leading to the diagnosis of an extragonadal non-seminomatous GCT, specifically a pure choriocarcinoma (100%).

The pulmonary lesions and extrapulmonary visceral metastasis in the liver classified the patient as having a poor prognosis in accordance with the International Germ Cell Cancer Collaborative Group (IGCCCG) criteria.^12,13^ The patient was assigned to stage IIIC according to both the Union for International Cancer Control (UICC) and the Lugano classification. At this time, the patient had an ECOG performance status of 0.

## Therapeutic interventions

**Figure 1.**
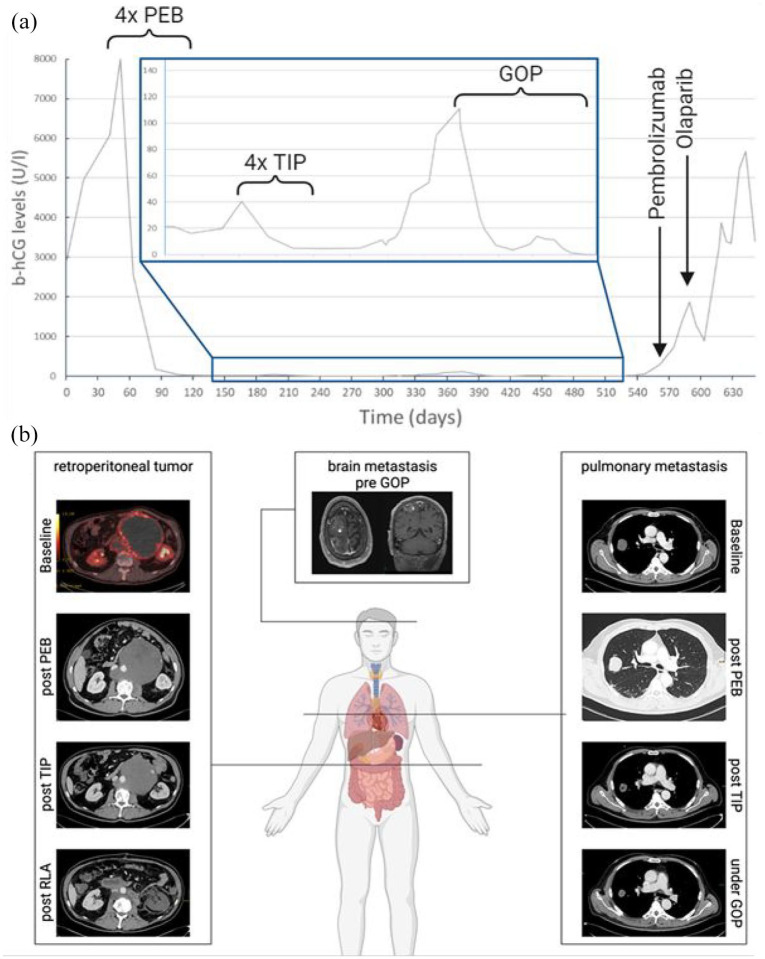
Schematic overview over time to show the treatment strategy with corresponding b-hCG course and metastatic tumor burden. (a) Timelines and schematic presentation of b-hCG course during treatment. Therapy regimes are indicated by curved brackets. First-line treatment with PEB over the course of four cycles. Salvage chemotherapy with high-dose TIP for four cycles. Third-line treatment with GOP. Finally, treatment with pembrolizumab and olaparib. (b) Cross-section imaging of the patient’s metastatic tumor burden. Retroperitoneal mass: baseline and after 1L treatment with PEB, after four cycles of high-dose TIP and post-RPLND. The tumor mass did not reduce in size and remained stable after PEB. After four cycles of TIP, the tumor slightly reduced in size. Also shows post-RPLND imaging. Brain metastasis: The largest of three brain metastases in the high right frontal lobe with perifocal edema and hemorrhage. Pulmonary metastasis: The largest of four lung metastases. Shown baseline and after PEB with slow growth under therapy. Significant size reduction after TIP and under GOP. Source: Created in Biorender. b-hCG, beta human chorionic gonadotropin; GOP, gemcitabine, oxaliplatin, and paclitaxel; PEB, cisplatin, bleomycin, and etoposide; RPLND, retroperitoneal lymph node dissection; TIP, paclitaxel, ifosfamide, and cisplatin.

According to IGCCCG classification,^12,13^ we initiated a treatment with four cycles of chemotherapy using cisplatin, etoposide, and bleomycin (PEB). A significant reduction in testicular tumor markers (Figure 1(a)) was observed, but they did not attain the upper limit normal of 2 U/l, with the minimum b-hCG level observed at 17 U/l, and thus confirming the b-hCG plateau. Consecutively, we initiated a salvage chemotherapy regimen, administering four cycles of paclitaxel, ifosfamide, and cisplatin (TIP). Post-TIP, b-hCG levels markedly declined and reached 5 U/l (Figure 1(a)), and restaging CT scans confirmed stable disease of the retroperitoneal tumor mass, and mixed response of the pulmonary and liver metastases. We performed a retroperitoneal lymph node dissection (RPLND) as a further therapeutic approach (Figure 1(b)). The histological analysis of the RPLND tissue specimen revealed 99% necrosis and 1% active tumor. We also excised the residual metastases in the right upper lung lobe, showing no signs of active tumor. Two months following surgery, the patient’s testicular tumor markers increased again (Figure 1(a)). The patient subsequently presented with symptoms of dyskinesia and headache. Imaging of the head showed three new metastases in the right frontal lobe (Figure 1(b)). Based on these new brain metastases with increasing tumor markers, we initiated a third-line chemotherapy regimen with gemcitabine, oxaliplatin, and paclitaxel (GOP) according to an open-label, multicenter phase II trial of the German Testicular Cancer Study Group.^14^ After three cycles of GOP, the clearly decreased brain metastases were surgically resected by craniotomy. Despite a decline in b-hCG levels, the GOP treatment had to be stopped after five cycles due to uncontrollable, therapy-induced, grade 3 thrombocytopenia.

Since the patient was not eligible for high-dose chemotherapy and subsequent stem cell transplantation, we conducted molecular profiling to gain deeper genetic insights into the characteristics of the tumor (Table 1). NGS of tumor DNA indicated a high TMB, with a rate of 10 mutations per megabase (mut/Mb). The microsatellites in the tumor were found to be stable. gLOH was 13% slightly lower than the cutoff of 14%–16% used for most cancer types.^15^ A variant with uncertain significance (VUS) in the ATM gene was found in 23% of the sequencing reads. An overview of genes tested with indeterminate results is presented in Supplemental Table 1. Based on the high TMB, borderline gLOH, and the detection of an ATM VUS, our local molecular tumor board decided on treatment with pembrolizumab 200 mg every 3 weeks together with olaparib 400 mg twice daily. The reporting of this study conforms to the Consensus-based Clinical Case Reporting (CARE) guideline development (Supplemental Figure 1).^16^

**Table 1.** Overview of molecular profiling.

Molecular profiling	Result
Microsatellite instability	Stable
Tumor mutational burden	High (10 mut/Mb)
Genomic LOH	Low (13% of tested genomic segments exhibited LOH)
ARID2	NM_152641.3: c.3184C>T (p.Q1062*); C5; VAF: 28%
BCOR	NM_017745.5: c.4395C>A (p.C1465*); C5; VAF: 51%
CIC	Deletion
ATM	NM_000051.3: c.2159G>A (p.R720H); VUS; VAF: 23%
NTRK3	NM_002530.3: c.2380C>A (p.Q794K); VUS; VAF: 54%

Mutations in chromatin remodeling and DNA damage response and repair genes, specifically a PV in Exon 15 of the ARID2 gene, precisely at pQ1062, with a VAF of 28%, another PV was identified in Exon 11 of the BCOR gene at pC1465, displaying a VAF of 51% and a variation in Exon 14 of the ATM gene at pR720H with a VAF of 23%. The sequencing for copy number aberration also uncovered a deletion of the CIC protein.

CIC, capicua; LOH, loss of heterozygosity; PV, pathogenic variant; VAF, variant allele frequency.

One month after GOP discontinuation, we initiated treatment with pembrolizumab since a further increase in tumor markers was observed. At this time, no significant immune-related adverse events were recorded. Two weeks following the first pembrolizumab cycle, we started administering olaparib. Shortly after beginning the olaparib treatment, b-hCG levels halved over a course of 3 weeks (Figure 1(a)). In summary, treatment with pembrolizumab and olaparib resulted in a notable decrease in tumor markers, occurring with the 3-week intervals.

Ten weeks following the initiation of combined therapy with pembrolizumab and olaparib, the patient succumbed to multi-organ failure due to immune-associated pneumonitis with a fatal outcome.

## Discussion

GCTs are highly responsive to platinum-based treatments, leading to successful outcomes in more than 80% of cases irrespective of metastatic disease. Two typical features of GCTs are related to cisplatin sensitivity: insufficient DNA repair of cisplatin-induced DNA damage, and a hypersensitive apoptotic response.^17^ However, approximately 10% of the remaining patients experience progression after salvage chemotherapy, developing resistance to platinum-based therapeutics.^4^ Patients relapsing within 4–8 weeks after platinum-based therapy, or who are progressing despite platinum-based therapy, as well as those relapsing shortly after high-dose chemotherapy, are defined as cisplatin-refractory according to the guidelines.^18^

Cisplatin-refractory disease presents a significant clinical challenge in identifying further effective therapies. The resistance to platinum-based chemotherapy and the lack of proven alternative options often result in a poor prognosis, necessitating further translational research. Moreover, the biological mechanisms behind this resistance are not completely understood, but they likely involve multiple, simultaneous processes that contribute to the emergence of resistant tumor cells. Treatment strategies that target multiple pathways simultaneously are likely to be more effective than single-agent therapies in overcoming this resistance.^19^

The testes have an immunologically specific environment, which is important to defend the germ cells.^20^ Previous studies showed that uniquely B cells and dendritic cells were present in testicular tumors. In addition, the presence of a pro-tumorigenic environment was suggested, due to increased transcripts of a large variety of cytokines and chemokines.^21^ It is furthermore known that testicular tumors have a higher infiltration of programmed cell death protein 1 (PD-1) expressing cells compared to normal testicular tissue, and both seminomas and nonseminomas express high levels of PD-L1 receptors.^22^ Besides this, many testicular tumors show hypervascularity, which, in turn, can support intratumoral immune cell migration.^23^ Because of the above, it is fair to say that testicular cancers have an active immune microenvironment, leading to the rationale of immunotherapy as a promising alternative therapy. Furthermore, based on the phase II KEYNOTE-158 trial, a high TMB has been associated with being a predictive biomarker for pembrolizumab treatment response.^24^ Therefore, we have selected pembrolizumab as part of the therapy strategy of our patient.

ICIs as a monotherapy are not likely to be accepted as an alternative therapy in treatment-refractory GCTs. Two phase II trials investigating the role of avelumab or pembrolizumab as monotherapy failed to achieve their primary endpoints regarding activity in resistant GCTs.^25,26^ Furthermore, different trials looked into the efficacy of various combination immunotherapy strategies. A phase II trial investigating the role of durvalumab alone, or in combination with tremelimumab also showed only limited outcomes, suggesting that dual immunotherapy in GCTs is only effective in highly selected cases.^27^ Although ICIs show insufficient efficacy in GCT, therapeutic combination approaches of immunotherapy with other targeted therapies, creating a possible enhanced synergy were proposed in the research community.

Currently, phase II trials are evaluating the potential of PARPis in refractory GCTs, either as a single agent (NCT02533765) or combined with gemcitabine and carboplatin (NCT02860819).^28^ Previous in vitro data confirmed that olaparib sensitized GCT cells to cisplatin treatment.^8^ Importantly, cells resistant to either cisplatin or PARPi monotherapy are highly sensitive to combination therapy, caused by genomic rearrangements, resulting in an increased load of DNA damage.^29^ This could be one more reason why PARPis could play a role as combination therapy with platinum-based chemotherapy in the salvage setting.^12^

The combination of PARPis with immunotherapy has also been investigated in different cancer types, with promising results.^30^ While many studies include the criteria of mutations in the *BRCA* genes,^31–33^ other preclinical studies suggest a synergetic use of PARPi together with immunotherapy even without considering the BRCA status.^30,34,35^ Unfortunately, not all clinical trials combining pembrolizumab with olaparib were successful. The phase III KEYLYNK-010 trial giving this combination to metastatic castration-resistant prostate cancer (mCRPC) patients showed no improvement in progression-free or overall survival.^36^ However, it has to be mentioned that they did not perform biomarker analysis (e.g., *BRCA* alterations) before selecting patients, which actually is commonly done in prostate cancer patients receiving olaparib.^37^ A systematic review and meta-analysis from Gazzoni et al., looking at the safety and efficacy of combining PARPi with ICI in mCRPC patients showed that these combinations mainly benefit patients with DDR mutations, such as *BRCA* alterations.^38^ Other mutations in the DDR pathway, like *ATM*, have also been associated with effective PARPi treatment^39^ and induction of PD-L1 expression.^40^ In our patient, we did not find any mutations in the *BRCA* genes, but a borderline gLOH, which would normally not be an indication for PARPi. However, this combinational treatment combining pembrolizumab with olaparib was selected as the last option for this patient who has exhausted all other conventional treatment options. A phase II trial combining pembrolizumab with olaparib in advanced cholangiocarcinoma patients, who failed or progressed on first-line therapy, showed to be a safe treatment option.^41^

Whether the observed therapeutic effects were caused by pembrolizumab or olaparib alone or by a combinative or synergistic effect of both remains speculative in this study. While there are available biomarkers predicting response to ICI or PARPi alone, there is a need to evaluate markers for this combinational therapy. Furthermore, more research is needed to investigate whether borderline TMB and borderline gLOH are possibly sufficient to be predictive of this combination therapy. However, due to the amount of background literature supporting this experimental treatment strategy, we have chosen this setting of immunotherapy in combination with olaparib for this patient, where all other standard-of-care treatments had already failed.

## Conclusion

Molecular profiling may hold considerable potential to introduce a personalized therapeutic approach also in GCT patients. However, clinical trials evaluating PARPi either alone or combined with chemotherapy have shown marginal efficacy in patients with cisplatin refractory GCT, and mixed results in different other cancer types. Thus, it is imperative to pursue further research with respect to evaluating the therapeutic efficacy of PARPi in combination with other targeted therapies or immunotherapy also even more in the front lines of therapy.

## Supplemental Material

sj-docx-1-tau-10.1177_17562872251322648 – Supplemental material for Pembrolizumab and olaparib in a cisplatin-refractory testicular cancer patient with a high TMB: first case report Supplemental material, sj-docx-1-tau-10.1177_17562872251322648 for Pembrolizumab and olaparib in a cisplatin-refractory testicular cancer patient with a high TMB: first case report by Nils C. H. van Creij, Gerald Klinglmair, Leonhard Gruber, Antonia Partl, Alain G. Zeimet, Frédéric R. Santer, Simon Schnaiter, Roman Mayr, Felizian Lackner, Zoran Culig, Andreas Seeber and Renate Pichler in Therapeutic Advances in Urology

## Declarations

**Ethics approval and consent to participate:** The patient gave written consent to use his medical data for research and publication.

**Consent for publication:** The patient gave written consent to use his medical data for research and publication.

**Availability of data and materials:** Data are available upon request.
