# Supplementary material for: Pembrolizumab and olaparib in a cisplatin-refractory testicular cancer patient with a high TMB: first case report
Source: Ther Adv Urol. 2025 Mar 14;17:17562872251322648. doi: 10.1177/17562872251322648 (PMC11907546; doi:10.1177/17562872251322648)

**Supplementary data**

**Material and Methods**

**Tumor Mutational Burden (TMB)**

TMB analysis was performed based on NGS analysis from genomic DNA isolated from a formalin-fixed paraffin embedded tumor sample using the Illumina platform. TMB is calculated using nonsynonymous, in-frame indel, and frameshift indel mutations that have not been previously reported as germline alterations in the Genome Aggregation Database (gnomAD) and dbSNP151 or as common benign variants identified by Caris geneticists. The cutoff of 10 mutations/megabase was established in non-small-cell lung cancer (NSCLC) and its applicability towards other tumor types has not been established at this time. Caris Life Sciences is a participant in the Friends of Cancer Research TMB Harmonization Project.

**Microsatellite instability (MSI)**

MSI by NGS (MSI-NGS) is measured by the direct analysis of known microsatellite regions sequenced in the Caris molecular intelligence (CMI) NGS panel. To establish clinical thresholds, MSI-NGS results were compared with results from over 2,000 matching clinical cases analysed with traditional, PCR-based methods. Genomic variants in the microsatellite loci are detected using the same depth and frequency criteria as used for mutation detection. Only insertions and deletions resulting in a change in the number of tandem repeats are considered in this assay. Some microsatellite regions with known polymorphisms or technical sequencing issues are excluded from the analysis. The total number of microsatellite alterations in each sample are counted and grouped into three categories: High (≥116 MSI loci altered), Equivocal (113-115 MSI loci altered) and Stable (≤112 MSI loci altered).

**Loss-of-heterozygosity (LOH)**

In order to calculate genomic LOH, the 22 autosomal chromosomes are split into 552 segments and the LOH of single nucleotide polymorphisms (SNPs) within each segment is calculated. Caris whole exome sequencing (WES) data consist of approximately 250k SNPs spread across the genome. SNP alleles with frequencies skewed towards 0 or 100% indicate LOH (heterozygous SNP alleles have a frequency of 50%). In this assay, a segment is determined to have LOH if the average SNP variant frequency is skewed more than ± 15% from the heterozygous frequency of 50% (p-value < 0.02 after correction vs. a negative control). The final call of genomic LOH is based on the percentage of all 552 segments with observed LOH (High ≥ 16%, Low < 16%; if fewer than 3,000 SNPs can be read, the test is reported as Indeterminate). A normal epithelial ovarian genome (NA12878), which has no non-polymorphic variants, gene fusions or other cancer hallmarks, is used as a negative control. Segment sizes range from 2-6 Mb, depending on segment proximity to the centromeres or telomeres. 99% of segments are at least 5Mb. Segments excluded from the calculation of genomic LOH include those spanning ≥ 90% of a whole chromosome or chromosome arm and segments which are not covered by the SNP backbone and the WES panel. The 250k SNPs consist of 200K from exonic regions and 50K from intronic regions, with a minimum of 17 SNPs per Mb of genome sequence.

**Next Generation Sequencing (NGS) Methods**

NGS for WES: Direct sequence analysis was performed on genomic DNA isolated from a microdissected, formalin-fixed paraffin-embedded tumor sample using the Illumina NovaSeq 6000 sequencers. A hybrid pull-down panel of baits designed to enrich for more than 700 clinically relevant genes at high coverage and high read-depth was used, along with another panel designed to enrich for an additional >20,000 genes at lower depth. A 500Mb SNP backbone panel (Agilent Technologies) was added to assist with gene amplification/deletion measurements and other analyses. The performance of the MI Exome assay was validated for sequencing variants, copy number alteration, tumor mutational burden and micro-satellite instability. The test was validated to 50ng of input and has a PPV of 99% against a previously validated NGS assay. For human leukocyte antigen (HLA) genotyping, analytical validation demonstrated concordance >99% to a validated comparator method. MI Exome can detect variants with tumor nuclei as low as 20%, and will detect variants down to 5% variant frequency with an average depth of at least 500x. This test has a sensitivity to detect as low as approximately 10% population of cells containing a mutation in all exons from the high read-depth clinical genes and 99% of all exons in the 20K whole exome regions. MI Exome is currently validated to detect <44bp indels. The reference genome for the transcript ID is hg38 with hg19 liftOver calculations performed for the high read-depth gene panel. While the vast majority of exons in the exome are covered by the assay, technical constraints preclude the coverage of every exon. **Supplementary Table 2** shows the high read-depth genes with the most relevance to cancer, that have only partial exon coverage. For a complete list of what is covered, please contact Caris Customer Support. HLA results are not available in New York State.

**Supplementary Table 1| Genes tested with indeterminate results.**

| Genes Tested with Indeterminate Results |
| --- |
| ACVR1B |
| COL2A1 |
| ELOC |
| NOTCH3 |
| PIK3R2 |
| POLD2 |
| PRKACA |
| PTPN11 |
| RASA1 |
| STAG2 |
| TRIM28 |
| XRCC1 |
| BTK |
| DACH1 |
| MED12 |
| PIK3CB |
| PLCB4 |
| PRDM6 |
| PRKD1 |
| PTPRD |
| SMARCA2 |
| TRAF7 |

**Supplementary Table 2| Cancer relevant genes that have a high read-depth, but only partial exon coverage.**

| Cancer relevant, high read-depth genes with only partial exon coverage |
| --- |
| ARID1B |
| ASXL2 |
| CDH23 |
| CDKN1C |
| CHEK2 |
| CYP2D6 |
| DIS3L2 |
| EIF1AX |
| FAT3 |
| FLT4 |
| FOXO3 |
| HSP90AA1 |
| HSP90AB1 |
| KMT2C |
| MAGI2 |
| MAML2 |
| MDS2 |
| MLLT3 |
| NCOR1 |
| NOTCH2 |
| NSD3 |
| PDE4DIP |
| PMS2 |
| RAC1 |
| RAD52 |
| RANBP2 |
| RB1 |
| RHEB |
| RPL10 |
| RPL22 |
| SBDS |
| SET |
| SMC3 |
| SRSF3 |
| STAT5B |
| SUZ12 |
| TCEA1 |
| TOP3B |
| TSHZ3 |
| USP6 |
| ZFHX3 |

**Supplementary Figure 1| CARE checklist.**


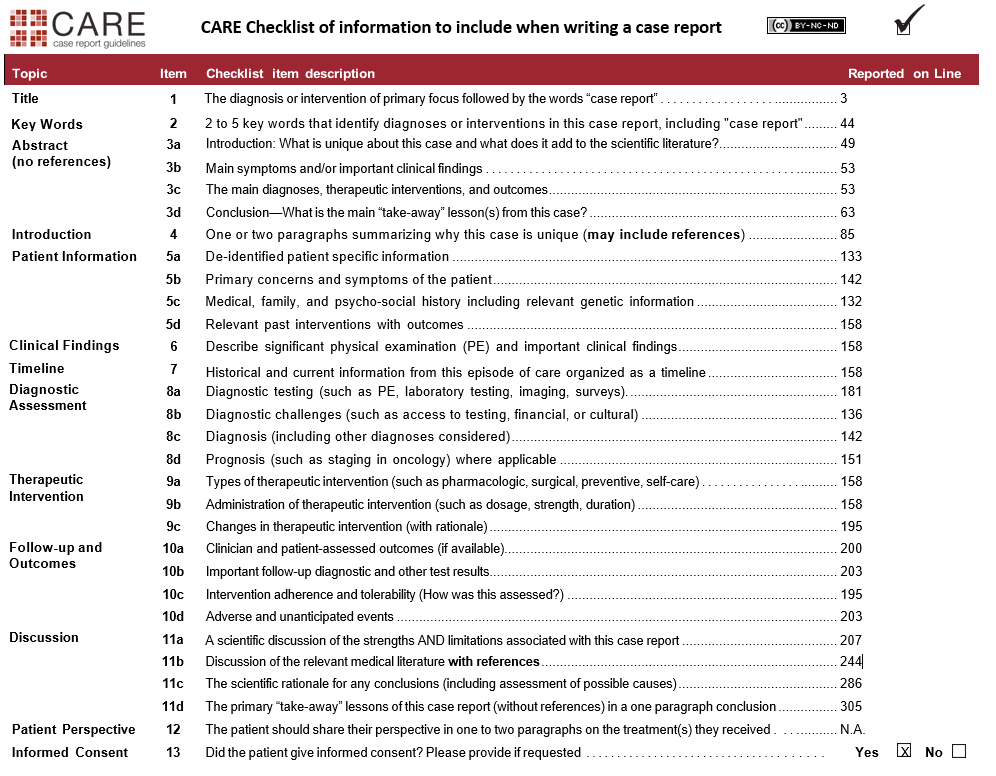

Supplement: sj-docx-1-tau-10.1177_17562872251322648 – Supplemental material for Pembrolizumab and olaparib in a cisplatin-refractory testicular cancer patient with a high TMB: first case report [file sj-docx-1-tau-10.1177_17562872251322648.docx]
